# Infections associated with eating seed sprouts: an international concern.

**DOI:** 10.3201/eid0505.990503

**Published:** 1999

**Authors:** P J Taormina, L R Beuchat, L Slutsker

**Affiliations:** Center for Food Safety and Quality Enhancement, University of Georgia, Griffin, Georgia 30223-1797, USA. taormina@cfsqe.griffin.peachnet.edu

## Abstract

Recent outbreaks of Salmonella and Escherichia coli O157:H7 infections associated with raw seed sprouts have occurred in several countries. Subjective evaluations indicate that pathogens can exceed 107 per gram of sprouts produced from inoculated seeds during sprout production without adversely affecting appearance. Treating seeds and sprouts with chlorinated water or other disinfectants fails to eliminate the pathogens. A comprehensive approach based on good manufacturing practices and principles of hazard analysis and critical control points can reduce the risk of sprout-associated disease. Until effective measures to prevent sprout-associated illness are identified, persons who wish to reduce their risk of foodborne illness from raw sprouts are advised not to eat them; in particular, persons at high risk for severe complications of infections with Salmonella or E. coli O157:H7, such as the elderly, children, and those with compromised immune systems, should not eat raw sprouts.


### Infections Associated with Eating Seed Sprouts: An International Concern

**Peter J. Taormina,* Larry R. Beuchat,* and Laurence Slutsker†**
*University of Georgia, Griffin, Georgia, USA; and †Centers for Disease Control and Prevention, Atlanta, Georgia, USA

Recent outbreaks of *Salmonella* and *Escherichia coli* O157:H7 infections associated with raw seed sprouts have occurred in several countries. Subjective evaluations indicate that pathogens can exceed $10^7$ per gram of sprouts produced from inoculated seeds during sprout production without adversely affecting appearance. Treating seeds and sprouts with chlorinated water or other disinfectants fails to eliminate the pathogens. A comprehensive approach based on good manufacturing practices and principles of hazard analysis and critical control points can reduce the risk of sprout-associated disease. Until effective measures to prevent sprout-associated illness are identified, persons who wish to reduce their risk of foodborne illness from raw sprouts are advised not to eat them; in particular, persons at high risk for severe complications of infections with *Salmonella* or *E. coli* O157:H7, such as the elderly, children, and those with compromised immune systems, should not eat raw sprouts.

With changing food production and eating habits, new pathogens and newly recognized vehicles of infection have emerged. Recent outbreaks of foodborne illness associated with eating fresh produce have heightened concerns that these foods may be an increasing source of illness (1). In the last decade, multiple outbreaks linked to raw seed sprouts have occurred in countries throughout the world (Table 1). Raw seed sprouts have become a popular food item in the United States; in a recent population-based survey, 7% of respondents had eaten alfalfa sprouts in the 5 days before the interview (2). We summarize the epidemiologic and microbiologic data from these outbreaks and review efforts to prevent sprout-associated illness.

### Sprout-Associated Outbreaks

Seed sprouts have been implicated as vehicles of transmission in outbreaks of foodborne illness (Table 1). One of the first reported outbreaks, in 1973, was associated with sprouts grown by using a home sprouting kit (3). Soy, mustard, and cress sprouts submitted by one person with gastrointestinal illness were found to contain large numbers of aerobic spore-forming bacteria. Bacteriologic examination of seeds in previously unopened sprouting kits revealed that the soy seeds were contaminated with *Bacillus cereus* in pure culture, while the mustard and cress seeds had *B. cereus* as a minor part of their flora. After germination, all the sprouts contained large numbers of the pathogen. Fecal specimens from patients were not analyzed for *B. cereus* because the laboratory that processed the samples did not consider it an enteric pathogen. Bacteriologic investigation revealed that during seed germination *B. cereus* proliferated to $>10^7$ per g of sprouts. In 1987, Harmon et al. (4) recovered *B. cereus* from 57% of commercially sold alfalfa, mung bean, and wheat seeds intended for sprout production.

### Salmonellosis

In 1988, raw mung bean sprouts were implicated in an epidemiologic study as the cause of an outbreak of *Salmonella* Saint-Paul infection in the United Kingdom (5). In addition, *S.* Virchow was isolated from samples of raw bean sprouts and was associated with seven cases of infection. Sprouts were produced from mung bean seeds imported mainly from Australia and Thailand. In a retail survey of mung bean sprouts in Thailand, several

Address for correspondence: Peter J. Taormina, Center for Food Safety and Quality Enhancement, University of Georgia, 1109 Experiment Street, Griffin, Georgia 30223-1797, USA; fax: 770-229-3216; e-mail: taormina@cfsqe.griffin.peachnet.edu.

Table 1. Reported outbreaks of illness associated with seed sprouts, 1973–1998

| Year | Pathogen | No. of culture-confirmed cases[a] | Location | Type of sprout | Likely source of contamination | Ref. |
|---|---|---|---|---|---|---|
| 1973 | *Bacillus cereus* | 4 | 1 U.S. state | Soy, cress, mustard | Seed | 3 |
| 1988 | *Salmonella* Saint-Paul | 143 | United Kingdom | Mung | Seed | 5 |
| 1989 | *S.* Gold-Coast | 31 | United Kingdom | Cress | Seed and/or sprouter | 7 |
| 1994 | *S.* Bovismorbificans | 595 | Sweden, Finland | Alfalfa | Seed | 8,9 |
| 1995 | *S.* Stanley | 242 | 17 U.S. states, Finland | Alfalfa | Seed | 10 |
| 1995-96 | *S.* Newport | 133[b] | >7 U.S. states, Canada, Denmark | Alfalfa | Seed | 11 |
| 1996 | *S.* Montevideo and *S.* Meleagridis | ~500 | 2 U.S. states | Alfalfa | Seed and/ or sprouter | 13 |
| 1996 | *Escherichia coli* O157:H7 | ~6,000 | Japan | Radish | Seed | 16 |
| 1997 | *E. coli* O157:H7 | 126 | Japan | Radish | Seed | 17 |
| 1997 | *S.* Meleagridis | 78 | Canada | Alfalfa | Seed | 15 |
| 1997 | *S.* Infantis and *S.* Anatum | 109 | 2 U.S. states | Alfalfa, mung, other | Seed | 14 |
| 1997 | *E. coli* O157:H7 | 85 | 4 U.S. states | Alfalfa | Seed | 18 |
| 1997-98 | *S.* Senftenberg | 52 | 2 U.S. states | Clover, alfalfa | Seed and/or sprouter | * |
| 1998 | *E. coli* O157:NM | 8 | 2 U.S. states | Clover, alfalfa | Seed and/or sprouter | * |
| 1998 | *S.* Havana, *S.* Cubana, and *S.* Tennessee | 34 | 5 U.S. states | Alfalfa | Seed and/or sprouter | * |

[a]The number of culture-confirmed cases represents only a small proportion of the total illness in these outbreaks, as many ill persons either do not seek care or do not have a stool culture performed if they do seek care.
[b]Includes only culture-confirmed cases in Oregon and British Columbia.
*Mohle-Boetani J., pers. comm.

serotypes of *Salmonella* were isolated from 8.7% of samples tested (6).

An outbreak of *S.* Gold-Coast in England and Wales in 1989 was associated with eating mustard cress sprouts grown from seed imported from The Netherlands. The outbreak serotype was isolated during routine sampling of cress sprouts from the factory 2 weeks before the outbreak occurred (7). Cultures of cress seeds did not yield the pathogen.

In Finland, eight sprout-borne *Salmonella* outbreaks occurred from 1980 to 1997 (8). In 1994, two large outbreaks of salmonellosis were linked to alfalfa sprouts (282 cases in Sweden and 210 cases in Finland) (9). Both outbreaks were caused by *S.* Bovismorbificans; the implicated sprouts were grown from Australian alfalfa seeds.

In 1995, a large international outbreak of *S.* Stanley infections in Finland and 17 states in the United States was caused by alfalfa sprouts grown from contaminated seeds (10). *S.* Stanley isolates from patients in Finland and the United States had an indistinguishable DNA pattern by pulsed-field gel electrophoresis (PFGE) and an unusual antimicrobial resistance pattern that was identical among outbreak strains but differed from *S.* Stanley strains isolated from nonoutbreak-related cases. Sprouts that caused the outbreaks in both countries were grown from seeds obtained from the same shipper in The Netherlands, suggesting the seeds were contaminated at some point during growing, harvesting, or processing.

In late 1995 and early 1996, outbreaks of salmonellosis in Denmark and Oregon and British Columbia, Canada, were associated with eating alfalfa sprouts contaminated with *S.* Newport (11). Patients in this multinational outbreak had eaten alfalfa sprouts grown from four separately numbered lots of alfalfa seeds. The seeds implicated in the North American outbreaks were shipped by the same Dutch firm implicated in the *S.* Stanley outbreak. A retrospective study determined that substantial increases in *S.* Newport infections occurred in Denmark and several states in the United States during the time that these seeds were likely to have been sprouted and eaten (11). PFGE patterns of *S.* Newport isolates from the Oregon and British Columbia outbreaks were indistinguishable from each other (11) and from isolates obtained during *S.* Newport outbreaks in late 1995 in Georgia and Vermont in the United States and in June 1995 in Denmark. Cultures of the implicated seeds yielded *S.* Newport (12).

In June 1996, the largest recorded sprout-associated outbreak in the United States occurred in California, resulting in >450 culture-confirmed cases of infection with *Salmonella* serotypes Montevideo and Meleagridis (13). The same strain of *S.* Meleagridis was isolated from patients and from alfalfa sprouts obtained from retail stores and the sprouting facility. Investigation at the sprouter revealed unsanitary sprouting practices and suboptimal employee hygiene. At the farm where the implicated alfalfa seed was grown, chicken manure was used to fertilize the field before planting. Horses grazed in adjacent fields, and their manure was collected and stored next to the alfalfa field.

An outbreak of *Salmonella* serotypes Infantis and Anatum, which occurred from February through June of 1997 in Kansas and Missouri, was associated with eating contaminated alfalfa sprouts produced by a local sprouter (14). On the basis of epidemiologic, traceback, and laboratory findings, the source of *Salmonella* contamination in this outbreak was determined to be alfalfa seeds.

In October 1997 in Alberta, Canada, an outbreak of *S.* Meleagridis infections was linked to eating alfalfa sprouts, and the outbreak serotype was isolated from retail product (15). During the same period, cases of *S.* Meleagridis infection with the same phage type occurred in persons who had eaten sprouts produced by sprouters in two other provinces but grown from the same alfalfa seed lot as the one implicated in Alberta.

In Northern California, in late 1997 and June 1998, two clusters of *S.* Senftenberg infections were associated with eating an alfalfa and clover sprout mixture; because the two types of sprouts were always mixed before sale, it was not possible to determine which type of seed was implicated (Mohle-Boetani J, pers. comm.). Cultures of clover and alfalfa seeds used to grow the implicated sprouts did not yield *S.* Senftenberg.

In May 1998, a cluster of *S.* Havana infections among patients in Arizona and California was linked to eating alfalfa sprouts (Mohle-Boetani J, pers. comm.). An outbreak of *S.* Cubana infections occurred from May to September 1998 among residents of Arizona, California, and New Mexico, also linked to eating alfalfa sprouts from the same grower implicated in the *S.* Havana outbreak. Alfalfa sprouts eaten by patients in both clusters were grown from the same seed lot, and cultures of seed from this implicated lot yielded *S.* Havana, *S.* Cubana, and *S.* Tennessee (Mohle-Boetani J, pers. comm.).

### Enterohemorrhagic *Escherichia coli* Infection

*Escherichia coli* O157:H7 infection has also been related to eating sprouts. In the world's largest reported outbreak of *E. coli* O157:H7 infections, which occurred in Japan in 1996, white (daikon) radish sprouts were epidemiologically linked to approximately 6,000 of the nearly 10,000 cases reported (16). The pathogen was not detected in cultures of implicated seeds. In the following year, white radish sprouts were again implicated in an outbreak of *E. coli* O157:H7 infection affecting 126 people in Japan (17).

In July 1997, simultaneous outbreaks of *E. coli* O157:H7 infection in Michigan and Virginia were linked by independent epidemiologic investigations with eating alfalfa sprouts grown from the same lot of seeds (18). Molecular subtyping by PFGE revealed that strains from outbreaks in both states were indistinguishable. The simultaneous occurrence of two geographically distinct outbreaks linked to the same lot of alfalfa seeds and caused by the same strain of *E. coli* O157:H7 strongly suggested that contaminated seeds were the source.

In June 1998, a cluster of *E. coli* O157:NM infections in Northern California and Arizona was associated with eating an alfalfa and clover sprout mixture produced by the same sprouter implicated in the *S.* Senftenberg outbreak (Mohle-Boetani J, pers. comm.). *E. coli* O157:NM isolates from the patients had indistinguishable PFGE patterns.

### Attempts to Control Microorganisms During Sprouting

Alfalfa and other types of seeds intended for sprouting are considered raw agricultural commodities. Seeds are harvested and transported from fields to sprouting facilities by methods similar to those used by the cereal grain and fresh produce industries. Grains, fruits, and vegetables may become contaminated with pathogenic microorganisms, e.g., *B. cereus, Salmonella*, or *E. coli* O157:H7, while growing in fields or orchards or during harvesting, handling, processing, and distribution (19,20). Alfalfa seeds generally contain $10^2$ to $10^5$ aerobic mesophiles per gram (21,22). Piernas and Guiraud (23) reported that the microflora on rice seed exceeded $10^7$ colony-forming units (cfu)/g. This naturally occurring population can rapidly increase during germination and sprouting, which is characterized by high moisture and a temperature generally in the range of 21°C to 25°C. Consequently, if seeds become contaminated with a pathogen, the sprouting process provides excellent conditions for its growth and distribution.

Populations of microorganisms on other seeds and sprouts have been studied. Potter and Ehrenfeld (24) detected non-O157 *E. coli* in 5 of 48 samples of mung bean seeds and mature bean sprouts, indicating possible fecal contamination. Alfalfa sprouts and bean sprouts in retail stores have been shown to contain microbial populations of $10^8$ to $10^9$ cfu/g (25); 6 of 23 retail samples of alfalfa sprouts contained $>10^5$ fecal coliforms per gram. Onion sprouts can contain $>10^9$ aerobic microorganisms per gram (20). Mung bean sprouts from restaurants may contain $>10^6$ cfu/g (26). Jaquette et al. (27) demonstrated that populations of *S.* Stanley in the range of $10^2$ to $10^3$ cfu/g can increase slightly during 6 hours of soaking, by approximately $10^3$ cfu/g during a 24-hour germination period, and by an additional $10^1$ cfu/g during a 72-hour sprouting stage, resulting in a 5- to 6-log overall amplification during the sprouting process. Pooled *Salmonella* serotypes inoculated onto mung beans and alfalfa seeds increased substantially during seed germination (21).

Growth characteristics of *E. coli* O157:H7 on radish sprouts have been studied. Itoh et al. (28) demonstrated the presence of *E. coli* O157:H7 not only on the surfaces but also in the inner tissues and stomata of cotyledons of radish sprouts grown from seeds inoculated with the bacterium. When radish seeds or radish sprout roots were soaked in a suspension of *E. coli* O157:H7, the edible parts (cotyledons and hypocotyl) became heavily contaminated (>7 log cfu/g) (29). Taormina and Beuchat (30) showed that *E. coli* O157:H7 inoculated onto alfalfa seeds reached $10^6$ to $10^7$ cfu/g within 48 hours after the sprouting process began. Populations on mature sprouts subsequently held at 9±2°C for 6 days remained essentially unchanged. Growth of *E. coli* O157:H7 to $10^7$ cfu/g of alfalfa sprouts has also been reported by Ingram et al. (31).

### Chemical Treatment as an Intervention

Numerous studies have been done to determine the effectiveness of a wide range of chemicals in killing pathogenic bacteria on seed sprouts and seeds intended for sprout production (Table 2). The efficacy of these chemicals as influenced by concentration, temperature, and time of exposure to contaminated seeds has been investigated. No single treatment has been demonstrated to reliably reduce populations of pathogens by more than approximately three logs.

Piernas and Guiraud (32) investigated different methods of disinfection of rice seeds. They observed $10^2$ to $10^3$ reductions in aerobic plate counts from rice seeds after treatment with 1,000 ppm NaOCl or 10,000 ppm (1%) $H_2O_2$ at room temperature. Ethanol was very effective in killing naturally occurring microorganisms, although it inhibited seed germination. Becker and Holzapfel (33) surveyed commercial prepackaged sprouts (alfalfa, lentils, wheat, peas, raphanus, sunflower, mung bean, and red radish) and found Enterobacteriaceae and pseudomonads to be the dominant groups of bacteria, with counts of $10^4$ to $10^5$ cfu/g. Washing sprouts in water did not remove bacteria; this treatment has been shown to reduce numbers of *E. coli* and *Salmonella* by no more than 1 log (24).

Treatment of bean sprouts with ozone has been shown to decrease microbial populations (34). Chlorine treatment, however, is ineffective in killing large numbers of naturally occurring microflora on seeds. Splittstoesser et al. (35) reported that treatment of sprouting mung beans with soak and rinse water containing 100 ppm chlorine reduced the natural microflora by <1 log; treatment of mature sprouts with 5,000 ppm chlorine resulted in a 2-log decrease (36).

The efficacy of chemicals in killing *Salmonella* on alfalfa seeds has been reported by several researchers. Jaquette et al. (27) evaluated chlorine and hot water treatments for their effectiveness in killing *S.* Stanley

Table 2. Control of microorganisms in seed sprouts, by type of treatment and treatment results

| Organism, origin | Treatment | Results of treatment | Ref. |
|---|---|---|---|
| Aerobic bacteria, rice seeds | 1,000 ppm NaOCl or 10,000 ppm $H_2O_2$ | $10^2$ to $10^3$ reductions in aerobic plate counts; germination inhibited | 32 |
| Enterobacteriaceae, pseudomonads, commercial sprouts | Washing in water | Ineffective in removing bacteria | 33 |
| Aerobic bacteria, mung bean sprouts | 100 ppm chlorine or 5,000 ppm chlorine | Reduced microflora by <1 log and 2 logs, respectively | 35 |
| *Salmonella* Stanley, alfalfa seeds | Chlorine and hot water | No reduction at low levels; reduction of *S.* Stanley achieved with 2,040 ppm chlorine | 27 |
| *Salmonella*, alfalfa seeds | 1,800 ppm $Ca(OCl)_2$ or 2,000 ppm NaOCl or 6% $H_2O_2$ or 80% ethanol | *Salmonella* populations reduced by >3 logs, but pathogen not eliminated | 37 |
| *E. coli* O157:H7, alfalfa seeds | 500, 1,000, or >2,000 ppm $Ca(OCl)_2$; 500 ppm acidified $ClO_2$; >100 ppm and 500 ppm acidified $ClO_2$; 30% or 70% ethanol; >1% $H_2O_2$; 8% $H_2O_2$ for 10 min; dry storage | Populations reduced but not eliminated; germination decreased; pathogen unaffected by dry storage at 5°C | 38 |
| *E. coli* O157:H7, alfalfa seeds at various stages of sprouting | 2,000 ppm NaOCl; 200 and 2,000 ppm $Ca(OCl)_2$; 500 ppm acidified $ClO_2$ | Populations substantially reduced but not eliminated | 30 |
| *S.* Stanley, alfalfa seeds | Heat, 54 to 71°C | 54°C for 5 min reduced population from 260 to 6-9 cfu/g; treatment for 10 min reduced viability of seed | 27 |
| *E. coli* O157:H7, alfalfa seeds and sprouts | Irradiation at >1.0 kiloGray | Pathogen controlled without affecting germination | 39 |

inoculated onto alfalfa seeds at populations of $10^2$ to $10^3$ cfu/g. Significant reduction ($p<0.05$) in population was observed when seeds were treated with 100 ppm chlorine for 5 or 10 minutes, and further reduction occurred after treatment with 290 ppm chlorine. Populations of $10^1$ to $10^2$ cfu of *S.* Stanley per g were reduced to undetectable levels (<1 cfu/g) after seeds were treated with 2,040 ppm chlorine solution. On the basis of these findings, in March 1996 the U.S. Food and Drug Administration recommended that sprout growers soak alfalfa seeds in 500 to 2,000 ppm chlorine solution for 30 minutes before sprouting. However, in none of the subsequent U.S. outbreaks listed in Table 1 was there documented evidence that this recommendation had been followed.

In another study, 10-minute treatment in solutions containing $Ca(OCl)_2$ or NaOCl at concentrations of 1,800 and 2,000 ppm chlorine, respectively, as well as 6% $H_2O_2$ or 80% ethanol, reduced *Salmonella* populations on alfalfa seeds by >3 logs (37) but did not eliminate the pathogen. Taormina and Beuchat (38) studied the efficacy of various chemical treatments in eliminating 2.0 to 3.2 $\log_{10}$ *E. coli* O157:H7 per g from alfalfa seeds and survivability of the pathogen on seeds during prolonged storage. Significant reductions ($p<0.05$) in population of *E. coli* O157:H7 on inoculated seeds were observed after treatments with 500 or 1,000 ppm chlorine [as $Ca(OCl)_2$] for 3 but not 10 minutes and with 2,000 ppm $Ca(OCl)_2$, regardless of pretreatment with a surfactant. Populations were reduced after treatment with 30% or 70% ethanol for 3 or 10 minutes, although germination percentage dramatically decreased. Treatment with 0.2% $H_2O_2$ for 3 or 10 minutes significantly ($p<0.05$) reduced populations of *E. coli* O157:H7 on alfalfa seeds, and the organism was not detected by direct plating after treatment with 1% $H_2O_2$. However, the pathogen was detected by enrichment in seed treated with 8% $H_2O_2$ for 10 minutes. The initial populations of 3 $\log_{10}$ cfu of *E. coli* O157:H7/g of dry seeds stored at 5°C remained relatively constant for 20 weeks.

Taormina and Beuchat (30) investigated the growth of *E. coli* O157:H7 on alfalfa seeds at various stages during sprouting as affected by NaOCl, $Ca(OCl)_2$, acidified $NaClO_2$, acidified $ClO_2$, $Na_3PO_4$, or $H_2O_2$. Spray application of 2,000 ppm NaOCl, 200 and 2,000 ppm $Ca(OCl)_2$, or 500 ppm acidified $ClO_2$ to germinated seeds significantly ($p<0.05$) reduced the population of *E. coli* O157:H7. None of the chemical treatments evaluated eliminated *E. coli* O157:H7 on alfalfa seeds and sprouts.

Application of heat to kill pathogens on alfalfa seeds has been investigated. Treatment of seeds containing approximately 260 cfu of *S.* Stanley per g at temperatures from 54°C to 71°C for 5 or 10 minutes was studied by Jaquette et al. (27). Treatment at 54°C reduced the number to 6 to 9 cfu/g. Treatment at 57°C for 5 minutes reduced populations to <1 cfu/g. Heating seeds at 54°C, 57°C, or 60°C for 5 minutes did not substantially reduce the viability of seeds; however, treatment at these temperatures for 10 minutes reduced viability from 96% (control) to 88%, 84%, and 42%, respectively. Although heat treatment appears to be effective in killing *S.* Stanley on alfalfa seeds, the range of temperatures that can be used is narrow, i.e., 57°C to 60°C for 5 minutes. Lower temperatures may not kill the pathogens, and higher temperatures or longer exposure time (10 minutes) decreased germination. Heating (55°C) alfalfa seeds containing 2.2 to 2.3 $\log_{10}$ cfu of *E. coli* O157:H7 per g in solutions containing up to 20,000 ppm chlorine, 1,200 ppm acidified sodium chlorite, 500 ppm acidified $ClO_2$, 5% $H_2O_2$, or 8% $Na_3PO_4$ for 3 minutes did not eliminate the pathogen (38).

The use of gamma irradiation to eliminate *E. coli* O157:H7 on alfalfa seeds and sprouts has been investigated (39). Studies at the U.S. Department of Agriculture have shown that doses approved for irradiating meat (which are higher than the 1.0 kiloGray dose allowed for fruits and vegetables) control *Salmonella* and *E. coli* O157:H7 on alfalfa sprouts. Both pathogens are more resistant to irradiation on dry seeds than on sprouts. At doses required to eliminate *E. coli* O157:H7, germination of seeds was not affected. These preliminary results need to be confirmed by other studies.

### Conclusions

Eating seed sprouts has been associated with numerous outbreaks in the United States and other countries, resulting in thousands of culture-confirmed illnesses; multiple pathogens have been involved, including *E. coli* O157, *B. cereus*, and many serotypes of *Salmonella*. Although most outbreaks have been associated with alfalfa sprouts, other raw seed sprouts have also been linked to illness.

Sprouts follow a complex path from farm to table that includes growing, harvesting, processing, and shipping of seeds, followed by sprouting and distribution of the finished product. Contamination can occur at any of these points in production and distribution. Measures that may help to reduce seed contamination include ensuring the use of properly treated manure as fertilizer on fields; using clean equipment to harvest, transport, and process seeds; and preventing contamination of seeds by rodents or other animals during processing, distribution, and storage. Some types of seeds used to produce sprouts for human consumption are also used to produce forage for animal feed, so these measures to reduce contamination may require changes in current agronomic, harvesting, and storage practices. At sprouting facilities, efforts must be made to ensure that good manufacturing practices are followed and that employees have access to adequate sanitary and handwashing facilities. Sprouters should be registered with the appropriate state and federal regulatory authorities to facilitate appropriate monitoring and inspection. To reduce the risk of sprout-associated foodborne disease, a comprehensive approach based on good manufacturing practices and principles of hazard analysis and critical control points needs to be implemented.

Compared with other fresh produce, sprouts pose a special risk because the sprouting process is a potent bacterial amplification step that occurs shortly before marketing and consumption. Pathogens can exceed $10^7$ per gram of sprouts during sprout production without adversely affecting the appearance of the product. Thus, technical approaches to ensuring sprout safety may require several steps to remove pathogens from seeds both before they are sprouted and during the sprouting process. The most effective chemical treatment currently available is soaking alfalfa seeds in 20,000 ppm active chlorine for at least 10 minutes before sprouting (38). However, this treatment may not be sufficient to eliminate the risk. Further research is needed to identify specific interventions, either applied alone or in combination with other chemical or physical treatments, to eliminate pathogens from contaminated seeds. The effort to address these research needs has resulted in an ongoing collaborative effort among industry, academia, and government, which provides a model example of interagency cooperation to prevent foodborne diseases (40,41). However, until effective measures to prevent sprout-associated illness are identified, persons who wish to reduce their risk for foodborne illness from raw sprouts are advised not to eat them; in particular, persons at high risk for severe complications of infections with *Salmonella* or *E. coli* O157:H7, such as the elderly, children, and those with compromised immune systems, should not eat raw sprouts (18,42).

Illness associated with eating sprouts and other fresh produce highlights the need for enhanced public health surveillance to detect foodborne outbreaks. Fresh produce such as lettuce, tomatoes, and seeds for sprouting may have complex and widely dispersed distribution patterns, as well as low or intermittent levels of contamination. Thus, outbreaks due to these items may be geographically diverse and have a low attack rate (1,43,44). Laboratory-based surveillance and subtyping of isolates from sprout- and produce-associated outbreaks are critical for recognition of these events and timely response. Subtyping of isolates, including serotyping and molecular typing such as PFGE, can help determine whether clusters of infections across several states are related (10), as well as to link geographically distinct outbreaks to a common source (18). At the national level, surveillance has been enhanced by a cluster-detection algorithm applied routinely to *Salmonella* serotype surveillance data to detect possible outbreaks (45). For *E. coli* O157:H7, a national electronic subtyping network has been established that will allow participating state public health laboratories and the Centers for Disease Control and Prevention to rapidly compare DNA PFGE patterns of *E. coli* O157:H7 strains with the patterns in a national database.

Some sprout-associated foodborne outbreaks have been international in scope, underscoring the importance of close communication and collaboration among nations to rapidly recognize and control such events (9-11). Successful response to international foodborne outbreaks has demonstrated the utility of a common language, such as *Salmonella* serotyping, for comparing strains from around the world (46). International surveillance networks such as Enternet (formerly Salm-Net) provide a forum for rapid exchange of surveillance data and notifications about outbreaks that may involve internationally distributed food products, including seeds intended for sprouts (10,47).

Peter Taormina is a graduate student in food science at the University of Georgia's Center for Food Safety and Quality Enhancement. His research interests include foodborne illness and the microbiologic hazards associated with fresh produce.

### References

1. Tauxe R, Kruse H, Hedberg C, Potter M, Madden J, Wachsmuth K. Microbial hazards and emerging issues associated with produce: a preliminary report to the national advisory committee on microbiologic criteria for foods. J Food Protection 1997;60:1400-8.
2. Centers for Disease Control and Prevention. FoodNet population survey atlas of exposures, 7/96–6/97, Atlanta: 1997.
3. Portnoy BL, Goepfert JM, Harmon SM. An outbreak of *Bacillus cereus* food poisoning resulting from contaminated vegetable sprouts. Am J Epidemiol 1976;103:589-94.
4. Harmon SM, Kautter DA, Soloman HM. *Bacillus cereus* contamination of seeds and vegetable sprouts grown in a home sprouting kit. J Food Protection 1987;50:62-5.
5. O'Mahony M, Cowden J, Smyth B, Lynch D, Hall M, Rowe B, et al. An outbreak of *Salmonella* Saint-Paul infection associated with bean sprouts. Epidemiol Infect 1989;104:229-35.
6. Jerngklinchan J, Saitanu K. The occurrence of salmonellae in bean sprouts in Thailand. Southeast Asian J Trop Med Public Health 1993;24:114-8.
7. Joce R, O'Sullivan DG, Strong C, Rowe B, Hall MLM, Threlfall EJ. A national outbreak of *Salmonella* Gold-Coast. Commun Dis Rep CDR Rev 1990:4:3-4.
8. Puohiniemi R, Heiskanen T, Siitonen A. Molecular epidemiology of two international sprout-borne *Salmonella* outbreaks. J Clin Microbiol 1997;35:2487-91.
9. Ponka A, Anderson Y, Sitonen A, deJong B, Jahkola M, Haikala O, et al. *Salmonella* in alfalfa sprouts. Lancet 1995;345:462-3.

10. Mahon BE, Ponka A, Hall WN, Komatsu K, Dietrich SE, Sittonen A, et al. An international outbreak of *Salmonella* infections caused by alfalfa sprouts grown from contaminated seeds. J Infect Dis 1997;175:876-82.
11. Van Beneden CA, Keene WE, Stang RA, Werker DH, King AS, Mahon B, et al. Multinational outbreak of *Salmonella enterica* serotype Newport infections due to contaminated alfalfa sprouts. JAMA 1999;281:158-62.
12. Aabo S, Baggesen DL. Growth of *Salmonella* Newport in naturally contaminated alfalfa sprouts and estimation of infectious dose in a Danish *Salmonella* Newport outbreak due to alfalfa sprouts. In: Program and abstracts of *Salmonella* and Salmonellosis '97; Nice, France; 1997;425-6.
13. Mouzin E, Werner SB, Bryant RG, Abbott S, Farrar J, Angulo F, et al. When a health food becomes a hazard: a large outbreak of salmonellosis associated with alfalfa sprouts. In: Program and abstracts of the 46th Annual Epidemic Intelligence Service Conference. Atlanta, GA:1997:15.
14. Glynn MK, Patrick S, Wuhib T, Neimann J, Flahert R, Boxrud D, et al. When health food isn't so healthy—an outbreak of *Salmonella* serotypes Anatum and Infantis infections associated with eating contaminated sprouts, Kansas and Missouri, 1997. In: Programs and abstracts of the 47th Annual Epidemic Intelligence Service Conference. Atlanta, GA:1998;16.
15. Buck P, Grimsrud K, Waters J, Cardinal R, Talbot J, Anand C, et al. Would you like a little *Salmonella* with your sandwich? In: Program and abstracts of the 47th Annual Epidemic Intelligence Service Conference, International Night. Atlanta, GA:1998.
16. Ministry of Health and Welfare of Japan. National Institute of Infectious Diseases and Infectious Disease Control Division. Verocytotoxin-producing *Escherichia coli* (enterohemorrhagic *E. coli*) infection, Japan, 1996–June 1997. Infectious Agents Surveillance Report 1997;18:153-4.
17. Gutierrez E. Japan prepares as O157 strikes again. Lancet 1997;349:1156.
18. Centers for Disease Control and Prevention. Outbreaks of *Escherichia coli* O157:H7 infection associated with eating alfalfa sprouts—Michigan and Virginia, June-July 1997. MMWR Morb Mortal Wkly Rep 1997;46:741-4.
19. Beuchat LR. Pathogenic microorganisms associated with fresh produce. J Food Protection 1996;59:204-16.
20. Beuchat LR, Ryu JH. Produce handling and processing practices. Emerg Infect Dis 1997;3:459-65.
21. Andrews WH, Mislivec PB, Wilson CR, Bruce VR, Poelma PL, Gibson R, et al. Microbial hazards associated with bean sprouting. Journal of the Association of Official Analytical Chemists 1982;65:241-8.
22. Prokopowich D, Blank G. Microbiological evaluation of vegetable sprouts and seeds. J Food Protection 1990;54:560-2.
23. Piernas V, Guirand JP. Microbial hazards related to rice sprouting. International Journal of Food Science and Technology 1997;32:33-9.
24. Potter AP, Ehrenfeld EE. Microbial loads of bean sprouts: coliform, *E. coli* and *Salmonella* [poster Q-243]. In the 98th General Meeting of the American Society for Microbiology; 1998 May 17-21; Atlanta, Georgia.
25. Patterson JE, Woodburn MJ. *Klebsiella* and other bacteria on alfalfa and bean sprouts at the retail level. Journal of Food Science 1980;45:492-5.
26. Sly T, Ross E. Chinese foods: relationship between hygiene and bacterial flora. J Food Protection 1982;45:115-8.
27. Jaquette CB, Beuchat LR, Mahon BE. Efficacy of chlorine and heat treatment in killing *Salmonella stanley* inoculated onto alfalfa seeds and growth and survival of the pathogen during sprouting and storage. Appl Environ Microbiol 1996;62:2212-5.
28. Itoh Y, Sugita-Konishi Y, Kusuga F, Iwaki M, Hara-Kudo Y, Saito N, et al. Enterohemorrhagic *Escherichia coli* O157:H7 present in radish sprouts. Appl Environ Microbiol 1998;4:1532-5.
29. Hara-Kudo Y, Konuma H, Iwaki M, Kasuga F, Sugita-Konishi Y, Ito Y, et al. Potential hazard of radish sprouts as a vehicle of *Escherichia coli* O157:H7. J Food Protection 1997;60:1125-7.
30. Taormina PJ, Beuchat LR. Behavior of enterohemorrhagic *Escherichia coli* O157:H7 on alfalfa sprouts during the sprouting process as influenced by treatment with various chemicals. J Food Protection 1999;62:850-6.
31. Ingram DT, Kantor MA, Meng J. Survival of *E. coli* O157:H7 during sprouting of inoculated alfalfa seeds. Int Assoc Milk Food Environ Sanit Ann Mtg, Prog and Abst Book, August 16-19, 1998, Nashville, TN. p. 36.
32. Piernas V, Guiraud JP. Disinfection of rice seeds to sprouting. J Food Sci 1997;62:611-5.
33. Becker B, Holzapfel WH. Mikrobiologisches risiko von fertgverpackten keimlingen und maßnahmen zur reduzierung ihrer mikrobiellen belastung. Archiv für Lebensmittelhygiene 1997;48:73-96.
34. Naito S, Shiga I. Studies on utilization of ozone in food preservation. IX. Effect of ozone treatment on elongation of hypocotyl and microbial counts of bean sprouts. Journal of the Japanese Society of Food Science and Technology 1989;36:181-8.
35. Splittstoesser DF, Queale DT, Andaloro BW. The microbiology of vegetable sprouts during commercial production. Journal of Food Safety 1983;5:79-86.
36. Moline HE, Kulik MM. Contamination and deterioration of alfalfa sprouts caused by a seedborne isolate of *Erwinia herbicola*. Journal of Food Quality 1997;20:53-60.
37. Beuchat LR. Comparison of chemical treatments to kill *Salmonella* on alfalfa seeds intended for sprout production. Int J Food Microbiol 1996;34:329-33.
38. Taormina PJ, Beuchat LR. Comparison of chemical treatments to eliminate enterohemorrhagic *Escherichia coli* O157:H7 on alfalfa seeds. J Food Protection 1999;62:318-24.
39. Gordenker A. Better detection methods needed for pathogens on fresh sprouts. Inside Lab Management, Association of Official Analytical Chemists International 1999;3:21-3.
40. Public meeting on sprout safety. U.S. Food and Drug Administration, Center for Food Safety and Applied Nutrition, Fresh Produce Subcommittee of the National Advisory Committee of Microbiological Criteria for Foods; 1998 Sep 28-29; Washington, D.C.
41. Pargas N. Sprout research to target 5-log reductions for *E. coli* O157:H7, *Salmonella*. Food Chemical News 1998; October 19:17.

42. Interim advisory on alfalfa sprouts. FDA. Washington, DC: August 31, 1998.
43. Tauxe RV. Emerging foodborne diseases: an evolving public health challenge. Emerg Infect Dis 1997;3:425-34.
44. Tauxe RV. New approaches to surveillance and control of emerging foodborne infectious diseases. Emerg Infect Dis 1998;4:455-6.
45. Hutwagner LC, Maloney EK, Bean NH, Slutsker L, Martin SM. Using laboratory-based surveillance data for prevention: an algorithm for detecting *Salmonella* outbreaks. Emerg Infect Dis 1997;3:395-400.
46. Tauxe RV, Hughes JM. International investigation of outbreaks of foodborne disease. BMJ 1996;313:1093-4.
47. Killalea D, Ward LR, Roberts D, de Louvois J, Sufi S, Stuart JM, et al. International epidemiological and microbiological study of outbreak of *Salmonella agona* infection from a ready to eat savoury snack – I: England, Wales, and the United States. BMJ 1996;313:1105-7.
